# An Investigation of Working Memory Profile and Fluid Intelligence in Children With Neurodevelopmental Difficulties

**DOI:** 10.3389/fpsyg.2021.773732

**Published:** 2022-03-18

**Authors:** Maria Sofologi, Vassiliki Pliogou, Eleni Bonti, Maria Efstratopoulou, Georgios A. Kougioumtzis, Efthymios Papatzikis, Georgios Ntritsos, Despina Moraitou, Georgia Papantoniou

**Affiliations:** ^1^Laboratory of Psychology, Department of Early Childhood Education, School of Education, University of Ioannina, Ioannina, Greece; ^2^Institute of Humanities and Social Sciences, University Research Centre of Ioannina (URCI), Ioannina, Greece; ^3^Department of Early Childhood Education, School of Humanities and Social Sciences, University of Western Macedonia, Florina, Greece; ^4^Department of Psychiatry, Faculty of Health Sciences, School of Medicine, Aristotle University of Thessaloniki, Thessaloniki, Greece; ^5^Department of Education, School of Education, University of Nicosia, Nicosia, Cyprus; ^6^Department of Special Education (CEDU), United Arab Emirates University (UAEU), Al Ain, United Arab Emirates; ^7^Department of Turkish and Modern Asian Studies, National and Kapodistrian University of Athens, Athens, Greece; ^8^Department of Early Childhood Education and Care, Oslo Metropolitan University, Oslo, Norway; ^9^Department of Informatics and Telecommunications, School of Informatics and Telecommunications, University of Ioannina, Arta, Greece; ^10^Department of Hygiene and Epidemiology, School of Medicine, University of Ioannina, Ioannina, Greece; ^11^Laboratory of Psychology, Section of Experimental and Cognitive Psychology, School of Psychology, Aristotle University of Thessaloniki, Thessaloniki, Greece; ^12^Laboratory of Neurodegenerative Diseases, Center for Interdisciplinary Research and Innovation (CIRI-AUTH), Balkan Center, Aristotle University of Thessaloniki, Thessaloniki, Greece

**Keywords:** developmental language disorder, high functioning autism, down syndrome, working memory function, fluid intelligence

## Abstract

The present study aims to evaluate the distinct patterns of working memory (WM) capacity of children with Developmental Language Disorder (DLD), High-functioning children with Autism Spectrum Disorder (ASD) and children with Down syndrome (DS). More specifically, the current study investigates the complex relationship of fluid intelligence and WM between 39 children with DLD, 20 H igh-functioning children with ASD, and 15 children with DS. All children were evaluated in different measures of Phonological Working Memory, Visual-spatial Working Memory whereas Fluid Intelligence was measured with Raven Progressive Matrices. The result analysis revealed a significant difference among the three groups, both among each function separately and the correlations among them, as well. The results revealed that the DLD groups and High-functioning ASD group exhibited a common picture or an overlap of performances in all Phonological and Visuo-spatial working memory measures, except Backward Digit Recall task. As for the DS group research findings revealed different and unique working memory patterns in comparison to DLD group and High-functioning ASD. Their differences have been studied and further conclusions have been drawn about the different patterns of working memory among the three clinical groups. The implications of these findings are discussed in light of support for learning. The common profile that characterize the two developmental conditions and the distinct pattern of working memory performance in DS group underlies the need for further research in the field.

## Introduction

Fluid Intelligence (FI) is a concept which describes the processes of abstract thinking and adaptability to new situations. More specifically, fluid intelligence is a cognitive skill which enables people to cope with a variety of problems, adapt to changing situations, and show flexibility in thinking. In parallel, General fluid intelligence (Gf) is a significant dimension of individual differences and is aligned with reasoning and problem-solving ability (Darki and Klingberg, 2015). It differs from Crystallized intelligence (Gc) since the latter is responsible for skills acquired by experience and not by immediate optimization and generalization procedures. Fluid intelligence and crystallized intelligence are facets of general intelligence (Hornung et al., 2011; Darki et al., 2015). Literature review reveals that, fluid intelligence is highly aligned and associated with frontal executive function (Duncan et al., 2000), attentional control and working memory capacity (Conway et al., 2002; Gray et al., 2003). Additionally, researchers emphasize the strong relationship of executive functions, monitoring, inhibition and simultaneously updating mental representations in the working memory resulting in the hypothesis that inductive thinking and strategies are part of fluid Intelligence (Miyake et al., 2000) and working memory capacity is a good prognostic factor for fluid intelligence (Heitz et al., 2006).

Working memory is a system which allows several pieces of information to simultaneously be held in mind in the course of the ongoing activities. Working memory is related to short-term memory, but also distinguishable from it. In specific working memory system is characterized by simultaneously storage and processing of information, whereas short-term memory systems specialize only in the temporary storage of information (Allen et al., 2014). This kind of system is clearly useful if you are trying to understand an utterance, where it may not be possible to process the beginning of the sentence fully until you have reached the end of it. It can be characterized as a mental workspace that can enhance daily cognitive actions which require not only processing but also storage, such as mental arithmetic and reading comprehension (Engle, 2010). Additionally, the capacity of working memory is constrained, and the processing demands and storage in the course of an ongoing cognitive activity will lead to gradually decrease of information from memory (Engle, 2010; Pulvermüller and Fadiga, 2010). There is a research consensus that memory and fluid intelligence apparently can be recognized as two significant factors contributing to learning process (Swanson, 1994).

In the sphere of the significant profile of working memory in dealing with higher-order cognitive functions, the research community have been aiming to study its influence not only in terms of the cognitive functioning in typically developing children but also in populations with neurodevelopmental disorders. Neurodevelopmental impairments involve difficulties in the growth and development process of the central nervous system or the brain (Peterburs et al., 2016). In specific, developmental impairments mirror a range of disorders of brain functions, which can have a negative impact on emotions, learning and memory ability, as well. Literature review reveals that children with neurodevelopmental disorders, like Autism Spectrum Disorder (ASD), Developmental Language Disorder (DLD), Tourette syndrome, etc., present different patterns of heterogeneity in working memory tasks and executive functions (Paul et al., 2016). ASD and other developmental disorders are closely aligned with weaknesses on cognitive tasks involving flexibility, inhibition, verbal reasoning, verbal memory, and language processing (Park et al., 2009; Nomi and Uddin, 2015). ASD is a polymorphous disorder with a heterogeneity profile with different impairments in language and in cognitive functions (Nomi et al., 2015). It is essential to mention that, although structural language impairment as part of a language interaction deficit is not a hallmark for an ASD diagnosis (according to DSM-5), it is highly aligned with ASD (Conway et al., 2002). In specific, literature review shows that approximately 63% of all children diagnosed with ASD have language impairment (Nomi et al., 2015).

A plethora of studies reveal contradictory findings concerning the working memory profile of children with ASD. There is skepticism about the exact nature of the working memory and executive deficits in populations with ASD (Miyake et al., 2000; Koshino et al., 2005; Minshew and Williams, 2007; Newcombe, 2016). Specifically, a series of researches have underlined the fact that children with ASD exhibit different profiles between verbal and visuospatial information processing in intelligence scales, such as Wechsler Intelligence Scale for Children (WISC), or Block Design and Object Assembly for verbal tasks (Miyake et al., 2000; Manolitsi and Botting, 2011). In specific, research findings reveal that children with ASD are characterized by difficulties in visual stimuli processing and in sustained attention (Wang et al., 2018) whereas they register better performances in verbal tasks and in visual perception stimuli (Operto et al., 2021; Pastorino et al., 2021). Additionally, some studies have found that children with ASD diagnosis showed better performances, than typical developing children in other visuospatial assessment tests, such as perceptual learning and visual searching (O’Riordan et al., 2001). They suggest that children with ASD tend to process low-level visual features into global structures, mirroring the hierarchical nature of the environmental stimuli (Tureck and Matson, 2012).

Along with the conflicting research evidence for the working memory profile of ASD, another neurodevelopment disorder which is characterized by diversity is DLD and its etiology is still not fully known (Alloway et al., 2004; Kemps, 2010). More specifically, the diagnostic characteristics of DLD involves language difficulties that are aligned with difficulties in daily language communication, and those language problems are unlikely to be resolved by the age of five (Montgomery, 1994). Moreover, DLD is associated with difficulties of both verbal working memory and memory capacity as well. Furthermore, research findings underlie the fact that children who have severe deficits in phonological loop capacity in DLD have been widely reported in studies measuring non-word repetition (Gathercole and Baddeley, 1990; Montgomery, 1995; Edwards and Lahey, 1998). Additionally, populations with DLD have also been found to be impaired on working memory tasks involving storage and processing of verbal material (Edwards et al., 1998) but not visuospatial information (Kemps, 2010). Finally, symptomatology of DLD diagnosis is characterized by heterogeneity; symptoms can be either expressive (e.g., syntax, vocabulary, phonology, and motor skills), receptive (i.e., comprehensive skills), or an amalgamation of the two.

In an attempt to clarify the working memory capacity patterns in different clinical populations with neurodevelopmental disorders, there is substantial evidence that individuals with Down syndrome (DS) also present a marked deficit of verbal short-term memory which exceeds their general difficulties with language (Kanno and Ikeda, 2002). Beginning at an early age, individuals with DS have impairments in adaptive functioning (Jarrold et al., 1999b) and specific cognitive domains, such as expressive language, and executive function, which are in excess of overall cognitive impairments. Groups with DS have been consistently found to be impaired on measures of verbal short-term memory relative to control groups, composed either of individuals with moderate learning difficulties of mixed etiology, or of younger typically developing children of compatible mental age (Jarrold et al., 1999b; Kanno et al., 2002). Their visuospatial short-term memory function, on the other hand, is typically appropriate to their mental age. The precise cause of this selective impairment of verbal short-term memory in DS is still not known. One possible reason is that individuals with DS have a particular problem with subvocal rehearsal, which plays a crucial role in actively maintaining phonological representations in short-term memory and preventing them from rapid decay (Jarrold et al., 1999a).

Another possible interpretation is that the low verbal memory performance associated with DS reflects inadequacies in the storage of phonological information in short-term memory (Schuchardt et al., 2010). Since intact phonological loop function is important for long-term learning of the sound structures of new words, it is likely that individuals with DS who have deficits of verbal short-term memory will acquire new vocabulary more slowly than it would be expected on the basis of their general cognitive abilities. There are studies conducted with children with DS and it is still not fully understood whether their difficulties emerge due to an impaired phonological loop system or as a byproduct of both phonological loop and cognitive deficits (Schuchardt et al., 2010). The current research attempts to synthesize findings of differential working memory impairments in order to make an important contribution to understanding the cognitive mechanisms which may give rise to domain-specific deficits.

## The Present Study

Under the theoretical aegis of the complex relationship between working memory capacity and fluid intelligence for children with High-functioning ASD, DLD and DS, these three groups share many common characteristics, such as difficulties in acquiring age-appropriate linguistic skills, while each group still has its unique strengths and weaknesses on tasks of working memory functioning. The importance of locating the similarities and differences of DLD, High–functioning ASD, and DS developmental conditions is critical in order to clarify the specific symptoms of each disorder and intervene accordingly. Despite research efforts, which have been made so far, the question is still open. In an attempt to illustrate the complexity of this multi-dimensional phenomenon, the present research aims to emphasize and clarify the distinct working memory patterns in these groups, as there has been contradictive evidence and interpretations from different research studies. More specifically, we attempted to shed light on the relationship between working memory function and fluid intelligence by comparing the performances of different neurodevelopmental groups. The advantage of such an approach is that it minimizes discrepancies due to test differences and allows for direct comparisons concerning performance across developmental disorders. As such, any differences in working memory skills could be attributed to a particular disorder. In this vein, the primary aim of the current study was to evaluate the profile of phonological working memory and the effect of fluid intelligence among the three groups of participants in phonological measurements. In specific, we hypothesized that children with DLD, High-functioning ASD, and DS will show different performances in all phonological working memory tests (Hypothesis 1). We expected that the DLD group will perform better in all phonological working memory tasks than the High-functioning ASD group and DS group, and also the High-functioning ASD group will register higher performances than the DS group (O’Riordan et al., 2001; Kemps, 2010; Tureck and Matson, 2012). According to the second hypothesis, we aimed to evaluate visuo-spatial working memory patterns among children with DLD, High–functioning ASD, and DS groups of participants in Visuospatial Working memory assessment tests. More specifically, we hypothesized that the three groups will show a different visual working memory profile (Hypothesis 2). Specifically, we expected that the DLD group will perform significantly better on visuo-spatial working memory tasks than the High-functioning ASD and DS group, whereas DS group will register higher performances in comparison with High-functioning ASD group on visuo-spatial working memory tasks (Miyake et al., 2000; Newcombe, 2016). Furthermore, taking into account that working memory and fluid intelligence are strongly related constructs, we aimed to investigate the possibility that the relationship between the working memory capacity (measured with both phonological and visuo-spatial WM tasks) and fluid intelligence could be different among the three different clinical groups and that these differences could be reflected on the process, short-term retention, and storage of verbal and visual information (Hypothesis 3). We hypothesize, especially, that the fluid intelligence will be positively correlated with all measures in DLD and High-functioning ASD groups but not in DS group reflecting a possible distinct cognitive profile (Jarrold et al., 1999b).

## Subjects and Methods

For the present study 74 children (54 boys and 20 girls) aged between 10 and 11 years old with different neurodevelopmental and genetic diagnoses were evaluated. More specifically, the first group of participants consisted of 39 children with DLD diagnosis (29 boys and 10 girls), and the second group consisted of 20 participants with High-functioning ASD diagnosis (15 boys and 5 girls). Finally, the third group consisted of 15 children with DS (10 boys and 5 girls). Demographic characteristics of all group participants are presented in Table 1. Participants were all Greek native, from mixed socio-economic backgrounds. The group with High-functioning ASD consisted of participants with a confirmed diagnosis of ASD from an authorized psychologist, or psychiatrist with the use of standard diagnostic protocols such as the Diagnostic Interview for Social and Communication Disorder (DISCO) and Autistic Diagnostic Interview/Observation Schedule (ADI-R/ADOS) following the criteria laid down by the DSM-IV for ASD. All participants came from special and state schools. The exclusion criterion was an additional diagnosis such as Attention Deficit Hyperactivity Disorder (ADHD), and other co-morbid diagnoses and hearing impairments. For the DLD group of participants, the selection criterion was an official diagnosis for DLD according to DSM-IV. In specific, the diagnostic criteria for DLD is unexplained and persistent difficulties with language acquisition including vocabulary, sentence structure, and discourse and lack of association with a variety of biomedical conditions such as brain injury, neurodegenerative conditions, genetic conditions or chromosome conditions, as well. Also, children with DLD diagnosis were excluded from the study if they had an additional diagnosis of ADHD, motor coordination disorder, and hearing impairments, as well. Finally, children with DS possessed formal diagnoses of the full Trisomy 21 DS karyotype, the most common form of the condition given by appropriate professionals using established diagnostic criteria, and were confirmed by parents/caregivers not to possess a co-morbid diagnosis of another developmental disorder, e.g., ADHD, ASD. Finally, two common exclusion criteria for all three groups were IQ scores under the average (IQ below 85) and any other history of psychiatric or medical conditions. Participants with lower levels of IQ were excluded. The children in the three clinical groups were recipients of special education services.

**TABLE 1:** Demographic characteristics of participants with developmental language disorder (DLD), High-functioning autism spectrum disorder (ASD), and down syndrome (DS).

Group participants	*N*	Age		Gender	
		Mean	S.D.	Boys	Girls
DLD	39	10.86	0.74	29	10
High functioning ASD	20	10.62	0.61	15	5
DS	15	10.57	0.74	10	5

### Assessment Instruments

Fluid intelligence (Gf) was evaluated by the Raven Colored Progressive Matrices Test (Raven et al., 2000). This scale is used to measure participants’ IQ and to ensure that no child presents intellectual disability among participants. For the assessment of phonological working memory the Backward Digit Recall was used (Georgas et al., 1997) and Sentence Recall Task (Pickering and Gathercole, 2001). Visual working memory was evaluated with the Visual Pattern Recall Test (VPT) (Della Sala et al., 1999) and Block Backward Test (Farrell Pagulayan et al., 2006). Both assessment tests are culturally neutral, non-verbal measures that include general shapes. This assessment tests were used in an attempt to evaluate the immediate visual retention of visual presented information in order to assess the direct involvement of the visual sketchpad.

### Raven’s Educational Colored Progressive Matrices

Raven’s Educational CPM/CVS consists of two subtests: the Colored Progressive Matrices (CPM) subtest, which is used for measuring non-verbal intelligence, and Crichton Vocabulary Scales (CVS) subtest, which measures verbal intelligence. The test is standardized in Greek population. In the present study, only the CPM subtest was used. It is considered an appropriate instrument for measuring non-verbal intelligence. Before its administration, each participant filled out a form concerning their demographic data. That is name, gender, school grade, examiner’s name, and date of birth (year, month, and day). During this test, the children were presented with a variety of schemas, a part of which is missing. The participants were offered six options and they had to choose which part better fills in the original schema. After the aforementioned introductory activity, 36 colored schemas of graduated difficulty, divided into 3 subscales of 12 each, were administered to them. Each correct answer scored 1 point. Therefore, each participant could collect 12 points in each subscale, that is, 36 points in total.

### Working Memory Measurements

#### Phonological Working Memory

##### Backward Digit Recall

The subtest of the Digit Recall test (verbal forward and backward recall) is part of the WISC-III standardized Greek version assessment tool (Georgas et al., 1997). The Digit Span Forward consists of eight complex gradient arithmetic sequences while the Digit Span Backward consists of seven. The examiner read a sequence of numbers and the participant had to repeat the same sequence in a reverse order (Digit Span Backward). For each pair of sequences, the first row is Attempt 1 and the second row is Attempt 2. Each arithmetic sequence is valued with one point. If the participant failed both attempts of the same pair, then the evaluation was stopped. Each question scores 2 points if the participant succeeds in both attempts of the question, 1 point if they successfully revoke only one of the two attempts of the question, and 0 points if they fail to recall the sequence of digits in both attempts at the question. T he sum of the correct answers for the backward digit recall is the sum of the correct answers. The maximum number of points in the backward digit recall, it is 14.

##### Sentence Recall Task

In the listening recall task, which is a phonological task, the child is presented with a series of spoken sentences by the examiner. The participant has to verify the sentence by stating “true” or “false,” and recalls the final word for each sentence in sequence. Every block consists of six sentences. Test trials begin with one sentence and continue with additional sentences in each block until the child is unable to recall three correct trials at a block. Participants are graded according to the total number of words successfully rehearsed (Maximum Score = 24, Minimum Score = 0). The mnemonic field score corresponds to the maximum number of words of the last field which was correctly recalled (Maximum score = 9, Minimum score = 0).

#### Visual Working Memory

##### Visual Pattern Recall

This assessment cognitive tool is used to measure visual short-term memory. The assessment consists of visual shapes (42 in total) which the participant is asked to reproduce immediately after the presentation. More specifically, the examiner presents a series of tabs in which there are combinations of squares, some of which are black and the others white. The participant is called upon to reproduce the previously presented image by tinting in the response protocol of the corresponding squares with those originally seen on each tab under consideration. The complexity of the test varies as it proceeds. The visual shapes vary in size from the smallest (2 × 2, i.e., two designed squares) to the largest shape (5 × 6 squares, that is, 15 designed squares). Each card is presented to the participant for 3 s and then removed from their field of view and then they are asked to reproduce in pencil and paper the shape which they have just seen. The answer booklet is placed in front of each participant, with the corresponding blank shapes, which are the same dimensions as the original visual shapes. The criterion for the process interrupting is the unsuccessful reproduction of two visual shapes in each field, regardless of the complexity of the design. Participants are graded in two ways: (a) according to the total number of shapes successfully reproduced (Maximum Score = 42, Minimum Score = 0).

##### Block Backward Test (Corsi Backward)

The Corsi Block Test consists of nine cubes perched on a rectangular wooden surface. This specific tests evaluates Central executive system according to Baddeley’s and Hitch (1974) model. Each cube is numbered from 1 to 10 (the numbers are visible only to the researcher). The researcher touches two or three, consecutive cubes at a time and the participant is asked to reproduce the sequence he or she has just seen. Touching each cube takes 1 s. The difficulty level fluctuates between fields, starting with one cube in the first field and reaching nine in the last one. Each field comprises a total of six attempts. Responses are scored 1 if they are correct and 0 if they are unsuccessful. This score gives the total number of correct answers. The final score corresponds to the sum of the correct answers (Maximum Score = 54, Minimum Score = 0). The mnemonic field score corresponds to the maximum number of cubes contained in the order of the last field which was correctly recalled (Maximum score = 9, Minimum score = 0).

## Procedure

All children participated in the study voluntarily. Each parent of every child received a file, which contained: (a) the information letter concerning the objectives of the research, as well as the contact details of the research supervisor, and (b) a consent form. All participants were examined individually and completed the study in three or four individual half-hour sessions in a quiet room. Practice trials could also be repeated to ensure comprehension of every task. All the participants were informed orally and in writing for the study and had the opportunity to ask questions. The participants could withdraw at any time, without giving a reason and without cost. Due to the specific type of the current research, demographic data such as age, gender, or occupation were selected. Since these are considered personal data, the European Union law that exists since May 28, 2018 was applied. According to the law, the use of sensitive personal data is allowed only due to research reasons. The study’s protocol followed the principles outlined in the Helsinki Declaration and was approved by the Scientific and Ethics Committee of the Greek Association of Alzheimer Disease and Related Disorders (68/15/05/2021).

### Data Analysis

Descriptive statistics of all three groups regarding their performances in all cognitive measures were calculated. Analysis of Variance (ANOVA) and Kruskal-Wallis *H* test, in case of non-parametric distribution of the data, were performed in order to examine if there were any statistically significant differences in Fluid Intelligence, Phonological Working Memory and Visuo-spatial working memory measures among the three different groups. To fully investigate the differences in the performances between the three groups on the Fluid Intelligence and Working memory tasks, multiple comparison *post hoc* tests (Tukey’s Honest Significant Difference (HSD) and Mann-Whitney test, in case of non-parametric distribution of the data, were performed.

To investigate possible correlations between the fluid intelligence, the phonological working memory assessment tests (Backward Digit Recall and Sentence Recall), and the visuo-spatial working memory tests (Visual Patterns and Corsi Block Backward Recall), for each group diagnosis separately, Pearson’s correlation coefficients were estimated. Furthermore, in order to study whether the relation between our participants is explained by group differences, or if there are differences within the groups of participants, we decided to proceed to a Linear Mixed Model (LMM) analysis (Singer and Willett, 2003). LMM technique provides the researcher with the opportunity to simultaneously study both within person (intra-individual) systematic change (level 1) and also between-person (inter-individual) differences (level 2) of the participants in different measures (Collins, 2006). Using this model, we can investigate the total among- and within person variance in the variables of interest. Using the within and among groups variances which emerged from the LMM, we, moreover, calculated the intra-class correlation coefficient (ICC), which indicates the proportion of the total variation of a variable that can be attributed to between-person differences. A high ICC (>0.10) implicit most of the differences that we observe across individuals on a variable are due to group differences (Hox, 2002; Collins, 2006). On the other hand, a low ICC suggests that the variation that we observe in a variable is due to individual differences within groups.

## Results

### Statistical Analysis

Table 2 presents the average and standard deviations of all three groups regarding their performances in all cognitive measures.

**TABLE 2:** Averages and standard deviations (SD) for three neurodevelopmental groups in all cognitive measures.

Group participants	DLD		High functioning ASD		DS	
	Mean	S.D.	Mean	S.D.	Mean	S.D.
*Phonological working memory*						
Backward digit recall	6.03	0.66	5.25	1.33	3.80	1.26
Sentence recall	17.33	3.20	22.60	2.98	16.28	4.16
*Visual working memory*						
Visual patterns	27.69	2.63	11.90	2.24	22.40	7.64
Corsi block backward	22.19	3.93	15.15	1.81	20.47	5.41
Fluid intelligence	31.23	2.31	22.10	3.47	26.53	4.40
Visuospatial WM (VP)	15.87	1.47	7.75	1.78	12.96	4.15
Central executive WM (CB)	12.98	4.95	10.00	2.65	13.80	4.81
Phonological WM (BDR and SR)	10.31	1.84	12.92	1.54	10.36	3.28

*VP, Visual Patterns; CB, Corsi Block Test; BDR, Backward Digit Recall; SR, Sentence Recall.*

In an attempt to evaluate the first hypothesis concerning the possible difference in the performance of the three groups (DLD, High-functioning ASD, and DS) in all phonological working memory tasks, an ANOVA was conducted with independent variables of the diagnosis from the three groups and the same applies to the dependent variables of their performance in all phonological working memory measurements. ANOVA indicates that there are statistically significant differences for all the variables of interest among the three groups in the phonological working memory measurements. Regarding the Backward Digit Recall task, statistically significant differences were observed between the three groups (*K-W H* = 31.8, *p* < 0.001). More specifically, the DLD group has statistically significant higher values than both the ASD and the DS group (mean values: 6.03 vs. 5.25, *p* = 0.034; mean values: 6.03 vs. 3.8, *p* < 0.001; respectively), and moreover, the High-functioning ASD group has statistically significant higher values than the DS group (5.25 vs. 3.80, *p* < 0.001). Regarding the Sentence Recall task, the mean values among the three groups the three groups differ on statistically significant degree [*F*_(2, 73)_ = 20.5, *p* < 0.001]. The High-functioning ASD group has statistically significant higher values than both the DLD and the DS group (22.6 vs. 17.33, *p* < 0.001; 22.6 vs. 16.28, *p* < 0.001; respectively). The findings confirmed the first research hypothesis.

**FIGURE 1:**
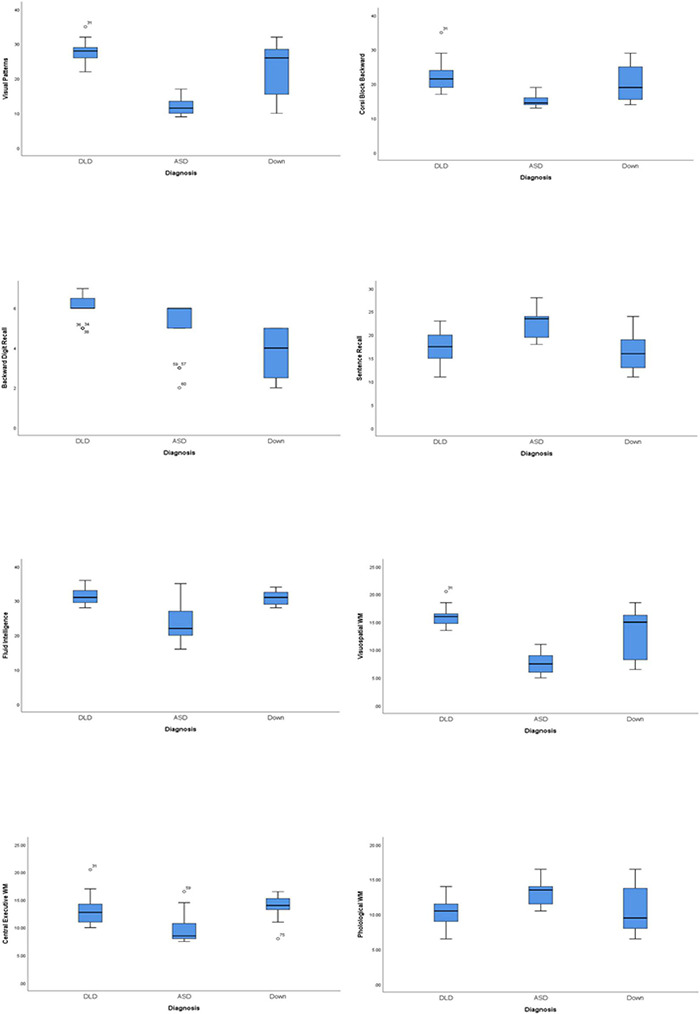
Boxplots for the graphically presentation of the differences for the fluid intelligence, the phonological working memory tasks, the visuo-spatial working memory measures, and the three general working memory variables central executive, phonological working memory, and visuo-spatial working memory among the three groups.

In the next part of the analyses, in an attempt to evaluate the second hypothesis according to which the performances of children with DLD, High-functioning ASD, and DS will differ in all visual working memory tasks, an ANOVA was performed. Analysis revealed that there are statistically significant differences for all the variables of interest among the three different groups. Regarding the Visual Patterns task, statistically significant differences were observed between the three groups (*K-W H* = 40.9, *p* < 0.001). More specifically, the ASD group has statistically significant lower values than both the DLD and the DS group (mean values: 11.90 vs. 27.69, *p* < 0.001; mean values:11.90 vs. 22.4, *p* < 0.001; respectively). Regarding the Corsi Block Backward test, statistically significant differences were observed between the three groups (*K-W H* = 32.2, *p* < 0.001). More specifically, the High-functioning ASD group has statistically significant lower values than both the DLD and the DS group (mean values: 15.15 vs. 22.19, *p* < 0.001; mean values: 15.15 vs. 20.47, *p* = 0.004; respectively). Regarding, the Raven’s Educational CPM, statistically significant differences were observed between the three groups (*K-W H* = 47.6, *p* < 0.001). More specifically, the DLD group has statistically significant higher values than both the ASD and the DS group (mean values: 31.23 vs. 22.1, *p* < 0.001; mean values: 31.23 vs. 26.53, *p* < 0.001; respectively), and moreover, the DS group has statistically significant higher values than the ASD group (mean values: 26.53 vs. 22.1, *p* = 0.009). Regarding the Visuospatial working memory, statistically significant differences were observed between the three groups (*K-W H* = 39.9, *p* < 0.001). More specifically, the ASD group has statistically significant lower values than both the DLD and the DS group (mean values: 7.75 vs. 15.92, *p* < 0.001; mean values:7.75 vs. 12.96, *p* < 0.001; respectively). Regarding the Central Executive working memory, statistically significant differences were observed between the three groups (*K-W H* = 20.4, *p* < 0.001). More specifically, the ASD group has statistically significant lower values than both the DLD and the DS group (mean values: 10.00 vs. 13.01, *p* < 0.001; mean values: 10.00 vs. 13.80, *p* < 0.001; respectively). Finally, regarding the Visuospatial working memory, the mean values among the three groups statistically differ significantly [*F*_(2, 73)_ = 10.9, *p* < 0.001]. More specifically, the ASD group has statistically significant higher values than both the DLD and the DS group (12.92 vs. 10.26, *p* < 0.001; 12.92 vs. 10.36, *p* < 0.001; respectively). The differences on the means of the variables of interest among the three groups are graphically presented in Figure 1.

### Relations Between Working Memory Measurements and Fluid Intelligence Among Three Groups

A full correlation matrix among measures is provided in Table 3. As shown in Table 3 fluid intelligence is positively correlated with both visuo-spatial working memory tasks (Visual Patterns and Corsi Block Backward) in DLD group and High-functioning ASD group. Furthermore, fluid intelligence measure did not show any statistically significant correlational relationship with the Backward Digit recall test in DLD group (*r* = 0.160) and High-functioning ASD (*r* = −0.165) whereas fluid intelligence is positive correlated with Sentence Recall test for DLD group (*r* = 0.348) and High-functioning ASD group (*r* = 0.505).

**TABLE 3:** Pearson’s coefficients for the correlations between the fluid intelligence, the phonological working memory tasks, the visuo-spatial working memory tasks, and central executive for each group diagnosis.

Group with DLD	Fluid intelligence	Visual patterns	Corsi block backward	Backward digit recall	Sentence recall	Visuospatial WM	CE WM	Phonological WM
Fluid intelligence	1							
Visual patterns	0.364*	1						
Corsi block backward	0.442**	0.510**	1					
Backward digit recall	0.160	−0.219	−0.192	1				
Sentence recall	0.348*	−0.019	−0.264	0.156	1			
Visuospatial WM	0.355*	0.986**	0.520**	−0.213	−0.022	1		
CE WM	0.440**	0.511**	0.995**	−0.167	−0.251		1	
Phonological WM	0.379*	0.023	−0.224	0.132	0.995*	0.022	−0.208	1
**Group with high-functioning ASD**								
Fluid intelligence	1							
Visual patterns	0.798**	1						
Corsi block backward	0.674**	0.689**	1					
Backward digit recall	−0.165	−0.079	0.114	1				
Sentence recall	0.505*	0.293	0.420	−0.530*	1			
Visuospatial WM	0.697**	0.537*	0.369	−0.591**	0.553*	1		
CE WM	0.449*	0.248	0.175	−0.901**	0.673**	0.805**	1	
Phonological WM	0.366	0.376	0.547*	−0.041	0.608**	0.173	0.083	1
**Group with DS**								
Fluid intelligence	1							
Visual patterns	0.431	1						
Corsi block backward	0.301	0.500	1					
Backward digit recall	0.319	0.829**	0.380	1				
Sentence recall	−0.414	−0.742**	−0.497	−0.735**	1			
Visuospatial WM	0.473	0.988**	0.502	0.835**	−0.757**	1		
CE WM	0.392	0.689*	0.556*	0.396	−0.349	0.685**	1	
Phonological WM	−0.315	−0.785*	−0.512	−0.849**	−0.965**	−0.801**	−0.346	1

The symbol ** is equal to p = 0.01 (2-tailed). The symbol * is equal to p < 0.05 (1-tailed).

On the other hand, correlation analysis revealed lack of statistical significant relationships between fluid intelligence and all the measures of phonological working memory (Backward Digit Recall *r* = 0.319, Sentence Recall *r* = −0.414) and visuo-spatial working memory measures (Visual Patterns Recall *r* = 0.431, Corsi Block Backward *r* = 0.301) for the group of participants with DS. The findings will be discussed further in the conclusion.

Additionally, for DS participants the performance on both the phonological working memory assessment tests (Backward Digit Recall and Sentence Recall) was found to be significantly related to visuo-spatial working memory (Visual Patterns *r* = 0.829). Furthermore, in the DS group matrix there was not any statistically significant relationship between the two visuo-spatial working memory tasks. On the contrary, the two visuo-spatial working memory tasks were found to be correlated with each other in DLD group and High-functioning ASD group. Furthermore, in DLD group and High-functioning ASD group both phonological working memory measures were not found to be correlated with any of the visuo-spatial working memory tasks. This finding may support the idea for common general cognitive mechanisms for DLD and High-functioning ASD and a different mechanism for on-line processing and temporarily preserved information in the DS group. Finally, in DS group and High-functioning ASD group the two phonological working memory measures (Backward Digit Recall and Sentence Recall) were found to have a statistically significant negative correlation to each other. It is essential to mention that due to the small number of participants we created three different variables of working memory measures in an attempt to evaluate and clarify possible relationships between WM tasks and Fluid Intelligence, three new variables emerged for WM. In specific, Corsi Block Test is the Central Executive variable, Sentence Recall and Backward Digit Recall is the Phonological Working memory variable and finally Visual Patterns test is Visuo-spatial working memory variable. A correlational analysis was performed between these three different Working memory variables and Fluid Intelligence. Results of the full correlation matrix among these three new Working Memory variables and Fluid Intelligence measures is provided in Table 3.

In order to further evaluate the relation between fluid intelligence and working memory tests in three different groups, confirmatory factor analyses (CFAs) were performed. CFA conducted in EQS Version 6.1 (Bentler, 2005) (using the maximum-likelihood (ML or ML ROBUST) estimation and performed on the three covariance matrices, which stemmed from each group of participants. More specifically, in an attempt to evaluate the relationships between Working Memory measures (Central Executive, Phonological Working Memory, and Visuo-Spatial Working Memory) and Fluid Intelligence, CFA with manifest variables was computed.

In the equations of the first CFA, the performance of the DLD group participants in Phonological Working Memory variable, Visuo-Spatial Working Memory variable, Central Executive measure, and R-Educational CPM was defined as measured variables loaded on a single latent variable of Fluid Intelligence. CFA verified the one-factor structure based on the three of the four observed variables for the DLD group [χ^2^(0, *N* = 39) = 0.00, *p* = undefined, NFI = 1.00]. NNFI, CFI, and RMSEA were not computed because the degrees of freedom were zero. This final model has been solved and should be considered as just-identified (see Brown, 2006). According to the suggestions of the initial CFA model’s Wald test, all the parameters of the model were statistically significant, except for the loading of one of the measured variables, namely, the loading of the Phonological WM measured variable (*p* = 0.53). Thus, in the final CFA model, we derived one factor of Fluid Intelligence (latent variable) that probably explains the variance of DLD students’ performance on Visuo-Spatial Working Memory measure, Central Executive measure, and R-Educational CPM.

Accordingly, a similar set of CFA was performed for the HF ASD group, in which the performance of the HF ASD group participants in Phonological Working Memory variable, Visuo-Spatial Working Memory variable, Central Executive measure, and Raven CPM was defined as measured variables loaded on a single latent variable of Fluid Intelligence. CFA verified the one-factor structure based on the three of the four observed variables for the HF ASD group [Satorra-Bentler scaled χ^2^(1, *N* = 17) = 3.56, *p* = 0.59, CFI = 0.95, RMSEA = 0.40 (CI90% 0.00–0.86). According to the suggestions of the initial CFA model’s Wald test, all the parameters of the model were statistically significant, except for the residual of one of the measured variables, namely the residual of the Visuo-Spatial Working Memory variable (*p* = 0.59) and the loading of one of the measured variables, namely, the loading of the Phonological WM measured variable (*p* = 0.10). Thus, in the final CFA model—showing the same pattern as DLD group—we derived one factor of Fluid Intelligence (latent variable) that probably explains the variance of HF ASD students’ performance on Visuo-Spatial Working Memory measure, Central Executive measure, and R-Educational CPM.

Finally, a similar CFA was performed for the DS group, in which the performance of the DS group participants in Phonological Working Memory variable, Visuo-Spatial Working Memory variable, Central Executive measure, and Raven CPM was defined as measured variables loaded on a single latent variable of Fluid Intelligence. However, the EQS 6.1 program (Bentler, 2005) warned that the disturbance of the residual of the Visuo-Spatial Working Memory measured variable was being held at the lower boundary (0.00) specified for the problem. The constraint of this parameter at lower boundary indicates a solution that is not acceptable: that the Visuo-Spatial Working Memory measured variable could be perfectly predicted from the latent variable of Fluid Intelligence. Therefore, we decided to perform path analysis and not CFA. In the equations of the path analysis, the performance of the DS group participants in Phonological Working Memory measure, Central Executive measure, and R-Educational CPM were defined as dependent variables (endogenous variable) and the performance in Visuo-Spatial Working Memory measure as an independent variable (exogenous variable). Path analysis fully verified the aforementioned model for the DS group [χ^2^(2, *N* = 15) = 0.25, *p* = 0.88, χ^2^/df = 0.12, CFI = 1.00, SRMR = 0.03 RMSEA = 0.00 (CI90% 0.00–0.24) (see Hu and Bentler, 1999; Brown, 2006). According to the suggestions of this model’s Wald test, all the parameters of the model were statistically significant, except for the correlation with the residuals of two of the dependent variables, namely the correlation of the residual of the Phonological Working Memory variable with the residual of the Central Executive variable (*p* = 0.11). Thus, according to this verified path model—showing a different pattern of what was found for DLD and HF ASD groups—we derived that Visuo-Spatial Working Memory measure (as an independent variable) is possible to explain part of the variance of DS participants’ performance on Phonological Working Memory measure, Central Executive measure, and R-Educational CPM. It should also be noted that the loading of the Phonological Working Memory variable to Visuo-Spatial Working Memory variable was negative.

Finally, in order to study whether the relation between our participants is explained by group differences, or if there are differences within the groups of participants, we decided to proceed to a LMM analysis (Singer and Willett, 2003). LMM technique provides the researcher with the opportunity to simultaneously study both within person (intra-individual) systematic change (level 1) and also between-person (inter-individual) differences (level 2) of the participants in different measures (Collins, 2006). Using this model, we can investigate the total among- and within person variance in the variables of interest. Using the within and among groups variances which emerged from the LMM, we, moreover, calculated the ICC, which indicates the proportion of the total variation of a variable that can be attributed to between-person differences. A high ICC (>0.10) implicit most of the differences that we observe across individuals on a variable are due to group differences (Hox, 2002; Collins, 2006). On the other hand, a low ICC suggests that the variation that we observe in a variable is due to individual differences within groups. The model ICC was significant for the performance on the fluid intelligence test and all the working memory tools. The model is presented in Table 4. The ICC percentages ranged between 38.3 and 71.9%, supporting that an important amount of the total variance of all the variables of interest, can be explained by inter-individual differences.

**TABLE 4:** Intra-class correlation coefficient (ICC) and Confidence Intervals for the performance on fluid intelligence test, the phonological working memory measures, and the visuo-spatial working memory tasks.

	ICC	CI
Backward digit recall	0.436	3.347–6.740
Sentence recall	0.383	13.640–23.832
Visual patterns	0.719	8.627–32.738
Corsi block backward	0.497	13.796–24.794
Fluid intelligence	0.638	21.668–32.936
Visuospatial WM	0.675	6.019–18.431
CE WM	0.298	9.247–15.286
Phonological WM	0.218	8.910–13.441

## Discussion

The present research aims to shed light on the working memory profiles of children with developmental disorders aiming to assess commonalities or differences of memory profiles in different populations with different developmental disorders. All these findings are very crucial for supporting children in developing not only their academic skills but also their social interaction competence. Working memory is of vital importance in our everyday social interrelationship, which is strongly aligned with storing and processing information continuously. In specific, the correlation matrices indicated that the DLD and High-functioning ASD group exhibit, in general, a common pattern of working memory profile. More specifically, concerning both groups important relations have been found between fluid intelligence, on the one hand, and the performance of visuo-spatial working memory and the Sentence Recall task which estimates phonological working memory *via* the verbal symbolic system, on the other. This correlation pattern verifies, in both groups, the known strong relation between fluid ability and working memory capacity (Colom et al., 2015) and it is possible to support the assumption of a possible existing comorbidity between High-functioning ASD and DLD (Norbury et al., 2004). Our findings are enhanced by previous research studies. Specifically, our analysis revealed that groups of High-functioning ASD and DLD are characterized by common characteristics mirroring a possible common profile between the two developmental disorders. This specific research finding is strengthen by the two path analysis for the DLD participants and High-functioning ASD. Results revealed the Fluid Intelligence is interpreted only from Visuo-spatial Working Memory and Central Executive but not from the Phonological Working Memory. These research data seems to be aligned with a serious of studies, emphasizing on the fact that children with ASD diagnosis, register similar performances with children with DLD diagnosis on different linguistic and working memory assessment tests (Norbury et al., 2004). Paradoxically, as far as the Backward Digit Recall is concerned, no relations were found with fluid intelligence in DLD and High-functioning ASD (Bishop et al., 2006). One possible interpretation for this finding could be that Backward Digit Recall task seems to measure phonological working memory *via* a numerical (digit) symbolic system of information, which is not mediated by semantic processing information and involves a modality-independent order coding system that is based only in numbers and demands number processing. Furthermore, it is characterized by dissimilar retrieval demands in comparison to the Central Executive measure the Corsi Block task; while participants tap different wooden blocks in the Corsi Block task, in the Digit Backward task the digits are not presented during the retrieval phase. As a result, it seems that phonological tasks are characterized by recalling both items and serial information, whereas visuospatial tests seem to demand the latter (Kjelgaard and Tager-Flusberg, 2001; Godfrey and Raitano, 2018). It is essential to point out that even though the Sentence Recall task and Backward Digit Recall are phonological tasks, there seems to be an asymmetry on the character of information that evaluate in on-line processing (lexical-semantic and digit-coding). Additionally, Backward Digit Recall involves executive functions and more specifically executive control (Kjelgaard et al., 2001; Colom et al., 2015; Godfrey et al., 2018).

As far as the performance of DS group’s fluid intelligence is concerned, it was not found to be correlated with phonological and visuo-spatial working memory measures reflecting a possible distinct cognitive profile in comparison with DLD and High-functioning ASD participants. One possible interpretation for the current finding is the small number of participants and this is a real limitation of the study. Possible different findings will be revealed with a larger sample of DS group. Moreover, the lack of correlations between fluid intelligence and phonological working memory measures can be interpreted by the fact that, researchers have suggested a differentiation between short term memory (STM) and working memory (WM) abilities in children with DS diagnosis. In specific, children with DS show a significant impairment in verbal and non-verbal WM assessment measures, but they do not register low performances in the non-verbal STM tasks despite profoundly impaired verbal STM abilities (Ritchie et al., 2015). This can be easily interpreted, due to the fact that our short-term memory system has limited capacity in terms of processing and as a result small amounts of information can be actively preserved and processed for a few seconds, whereas working memory involves temporary storage of limited amounts of information, and it specifically holds information that are necessary for the task that the individual carries out. Furthermore, working memory demands actively maintenance and attentional control while simultaneously processing information, avoiding distraction, and/or engaging in cognitive shifting, a trait that characterizes the Backward Digit Recall (Ritchie et al., 2015). This different pattern appears to be an important interpretation when considering interrelations between these two short-term memory systems and higher level cognitive skills. Additionally, literature review reveals that Working Memory is strongly aligned with intellectual abilities in typical development children but not always for clinical groups (Higo et al., 2014). Furthermore, children with DS consistently register worse performances than typical developing children on verbal WM tasks a performance that may well be under general genetic constraints (Georgiou and Spanoudis, 2021). Additionally, the lack of associations between fluid intelligence and all working memory measures may be associated with the fact that experimenters were giving an extra administration time processing and this can fade the cognitive relationship of fluid intelligence and working memory for the DS group (Georgiou et al., 2021). The lack of relations in DS may be supported by the idea that working memory difficulties do not reflect impairment to a distinct cognitive system, but rather could be impacted by specific genetic modular deficits that are characteristic of the disorder (Lanfranchi et al., 2015). Moreover, for the DS group the correlation matrix revealed that both Visuo-spatial working memory measures were not found to be related despite the fact that this specific relation is high, a finding that can be changed to a statistical significant positive relation if the number of participants increased. On the other hand, for the DS group the Visual Patterns test shows to be related with the Backward Digit Recall which is a modality-independent order coding system which is based only on numbers and demands number processing.

As far as the DS group and High-functioning ASD group are concerned, it is important to point the negative relation in Backward Digit Recall and Sentence Recall, whereas in the DLD group does not exist, a performance pattern that is aligned with typical developing children. We assume that for the High-functioning ASD group the negative relation in the Backward Digit Recall indicates difficulties in number processing and encoding in comparison with the Sentence Recall task. With respect to research findings children with ASD struggle in most of the verbal working memory tasks as these are linked to attentional problems and cognitive flexibility (Frith and Happé, 1998). They devote serious and sustained attention trying to store and process verbal information, rather than trying to store phonological information only. These deficits may reflect the multiplicity of cognitive skills which contribute to the two phonological tasks (Gathercole et al., 2006).

Additionally, results from the second hypothesis revealed that all groups differentiated in all visual working memory measurements. In specific, all groups attempted to manipulate and process visual information irrespective of the nature of the information to be remembered or mentally processed. A possible interpretation is that these children had difficulties manipulating their mental processing behavior and so tried to focus on the information in the first instance. As a result, their low working memory performances were a reflection of a lack of behavioral inhibition or social communication rather than a working memory deficit *per se* (Frith et al., 1998). Furthermore, research reveals that children with ASD and DS show better performances with simpler tasks across domains, such as simple attention, memory, language, or visuospatial tasks, than with more complex tasks such as skilled motor, complex memory, complex language, and reasoning domains (Hox, 2002; Norbury et al., 2004). Finally, the third hypothesis revealed that the structure of working memory varies among the three groups, based on the performance of working memory and fluid intelligence assessments. This research finding supports the theoretical idea that working memory and fluid intelligence are not isomorphic constructs (Mehta et al., 2004; Archibald and Gathercole, 2007; Mandy et al., 2018). In specific, for the DLD group performance literature review shows that although participants may register scores in the normal range for non-verbal intelligence tasks, they are sometimes as much as two standard deviations below their potential when compared with typical development children. This finding show that although language function is impaired in DLD participants, non-verbal intelligence may also be deficient, albeit to a lesser degree. This fact creates an alternative interpretation according to which a possible general low-level processing impairment can have an impact on a variety domains but to differing degrees and at different developmental periods. Also, there is a consensus among researchers in the last decades that confirm the hypothesis of a non-complete modal nature of the relationship between working memory and fluid intelligence. This theoretical approach is based on research findings according to which the correlations between tasks with overlapping content for working memory and fluid intelligence were higher than those for non-overlapping tasks. Additionally, for the performance of the DS group arises a specific question whether the environment is the same for individuals developing typically and those developing atypically. According to Karmiloff-Smith (2009) parents of toddlers with DS, tend to rapidly “veto” any overgeneralization, probably because they fear that the child with lower intelligence will never learn the correct term if allowed to overgeneralize. However, initial overgeneralization in the healthy child may actually encourage category formation, known to be subsequently impaired in the atypical case. Such unconscious assumptions about atypical development may lead parents to provide less variation in linguistic input, shorter sentences, and in general a less richly varied environment. These quite subtle changes in the child’s environment are likely to compound over time, in that the environment of the atypically developing child may increasingly differ from that of the typically developing child. This specific hypothesis can support the DS group differentiation of performances.

One limitation of the present study is the sample size for the High-functioning ASD group and DS group. It is important to highlight this parameter as a larger sample could reveal more accurate distinct working memory patterns. Furthermore, another limitation of the current study is the nature of the working memory tasks. By attempting to illustrate the working memory profiles of the three neurodevelopmental groups a proposal for future research will be the use of computerized assessment working memory tests for more accurate measurements. The neuropsychological instruments are proposed to be given through a computer in a controlled environment to measure more efficiently without the help of the examiner. The future purpose of studies evaluating the relationship of working memory and fluid intelligence should be the evaluation of cognitive profiles with Working memory Test Batteries and verbal and non-verbal intelligence, as well. Despite the restrictions that research can have, its findings effectively affect and strengthen the cognitive basis of the three diagnoses by providing a new insight into the cognitive profiles of children with DLD, ASD, and DS (Bennetto et al., 1996; Ackerman et al., 2005). Also, current research findings support the theoretical idea that human intelligence and working memory is not a state (i.e., not a collection of static, built-in modules handed down by evolution and that they can be intact or impaired). On the contrary, human intelligence is a dynamic process (i.e., the emergent property of dynamic multidirectional interactions between genes, brain, cognition, behavior, and environment). There is, therefore, a vital need for scientific studies on how having a developmental disorder subtly changes the social, cognitive, linguistic, and physical environment in which the atypically developing child grows up, which has important implications for intervention (Aron, 2007). Research, so far, cannot provide conclusions on the similarities and differences between the three disorders. Finally, researchers need to reconsider the notion of featural processing at the cognitive level. The need for more future research in the area is underlined, to provide evidence either to support or dismiss the assumption of a common etiology behind DLD, ASD, and DS.

## Conclusion and Implications

In conclusion, our results enhanced the assumption of an almost common symptomatology of the DLD and High-functioning ASD group of participants. On the other hand, DS group revealed a different profile which may originate from very different cognitive/brain causes. To conclude, the current study has provided research findings for skepticism in evaluating and comprehending the memory profiles of children with neurodevelopmental disorders by comparing their performance with each other. This research has also highlighted the importance of the relationship of fluid intelligence with working memory. In summary, the present study investigated the strengths and weaknesses of working memory in different developmental disorders. We believe that the distinct memory profiles associated with each disorder reflect the nature of their deficit to some degree. Working memory appears to be a secondary deficit, possibly driven by core deficits in language, behavior, or social difficulties. This corresponds with the view that a core impairment associated with particular developmental disorders can have a cascading effect on other cognitive skills. Additionally, one basic parameter that this study aims to emphasize is the fact that these children need to be given brief assessment tests, supporting them to learn different mnemonic strategies, which can help them manipulate and process information regularly. The major focus of an intervention future study should also be on enhancing their short-term memory system as a first intervention step and gradually helping them to manipulate tasks requiring simple processing, maintaining and storage of information. As a result, the brief short-term tasks can be transformed to cognitive basis in order to enhance and support working memory abilities. We know that a Full-Scale IQ consists of two major constructs, crystallized and fluid intelligence. We also know that crystallized intelligence is based on learned material. Hence, this would make a strong case for improvement of memory within a stimulating environment. This makes a strong case for the provision of support to children who function at a lower level of intellect as this may help improve their deficits (Baird et al., 2006). In parallel, phonological short-term memory deficits could be compensated by areas of strength in visuo-spatial short-term memory through the use of visual aids such as look-up tables (Baird et al., 2006). Conversely, difficulties in visuo-spatial short-term memory can be enhanced by relying on verbal strategies like rehearsal (Bonifacci, 2004). Where working memory deficits are present, the child will struggle to hold in mind and manipulate relevant material in the course of ongoing mental activities. Finally, under the aegis of the above mention research studies an essential goal of every research study with children’s population is to built up a positive school climate for the holistic development of all students (Sofologi et al., 2020).

## Data Availability Statement

The raw data supporting the conclusions of this article will be made available by the authors, without undue reservation.

## Ethics Statement

The studies involving human participants were reviewed and approved by the Greek Association of Alzheimer Disease and Related Disorders 68/15/05/2021. The patients/participants provided their written informed consent to participate in this study.

## Author Contributions

MS and GP: conceptualization. MS, VP, and GN: methodology and data curation. MS, DM, GP, and EP: validation and formal analysis. MS, GK, ME, VP, and GP: investigation. MS, VP, EB, and GN: resources. MS and ME: writing—original draft preparation. MS, DM, and GP: writing—review and editing. MS, GK, and EB: visualization. MS, VP, and GP: supervision. MS and VP: project administration. All authors contributed to the article and approved the submitted version.

## Conflict of Interest

The authors declare that the research was conducted in the absence of any commercial or financial relationships that could be construed as a potential conflict of interest.

## Publisher’s Note

All claims expressed in this article are solely those of the authors and do not necessarily represent those of their affiliated organizations, or those of the publisher, the editors and the reviewers. Any product that may be evaluated in this article, or claim that may be made by its manufacturer, is not guaranteed or endorsed by the publisher.
